# Myocardial strain features by 2D-STE during the course of fulminant myocarditis: Correlation with characteristics by CMR and clinical implications

**DOI:** 10.1097/MD.0000000000025050

**Published:** 2021-04-16

**Authors:** Houjuan Zuo, Haojie Li, Rui Li, Fei Ma, Jiangang Jiang, Chenze Li, Liming Xia, Hong Wang, Dao Wen Wang

**Affiliations:** a Division of Cardiology, Department of Internal Medicine, Tongji Hospital, Tongji Medical College, Huazhong University of Science and Technology, Wuhan; b Hubei Key Laboratory of Genetics and Molecular Mechanisms of Cardiologic Disorders, Wuhan, Hubei Province; c Department of Radiology, Tongji Hospital, Tongji Medical College, Huazhong University of Science and Technology, Wuhan, China.

**Keywords:** 2D speckle tracking echocardiography, acute myocarditis, cardiovascular magnetic resonance, fulminant myocarditis, global longitudinal strain, left ventricular function

## Abstract

Myocardial strain analysis by 2D speckle tracking echocardiography could determine the left ventricular function. Our purpose is to investigate the global longitudinal strain (GLS) changes during the course of fulminant myocarditis (FM) and evaluate their correlation with cardiac magnetic resonance (CMR).

Patients with clinical diagnosis of FM from June 30, 2017 to June 30, 2019 were screened prospectively. 18 survived patients (mean age 34 ± 18 years) who had two scans of transthoracic echocardiography and underwent CMR were included.

All patients had severely impaired left ventricular ejection fraction and GLS value at admission that improved significantly before discharge. The patients in the healed stage revealed elevated global native T1 and T2 relaxation time and extracellular volume fraction as well, which were 1408.3 ± 88.3ms, 46.56 ± 5.23ms, and 0.35 ± 0.09, respectively. GLS from the second transthoracic echocardiography in the healed stage correlated significantly with global native T1 relaxation time (r =-0.574, *P* = .013) and with extracellular volume fraction (r = -0.582, *P* = .011), but not global native T2 relaxation time (r = -0.31, *P* = .211) and not with late gadolinium enhancement mass (r = 0.084, *P* = .743). In comparison, GLS at admission were not correlated with CMR parameters of fibrosis and oedema in the healed stage.

GLS by 2D-STE may emerge as a new tool to monitor inflammatory myocardial injuries during the course of FM. FM in the acute healed stage has the presence of both chronic fibrosis and oedema which are correlated with GLS, but GLS at admission can’t predict the early recovery of myocardial inflammation.

## 1 Introduction

Fulminant myocarditis (FM) has been described in clinical settings as a clinical syndrome with rapid deterioration of hemodynamic status or heart function. FM patients often have dramatic presentation and a large proportion of them may die from cardiogenic shock or sudden death in the acute phase as reported.^[1–5]^ In contrast, some FM patients exhibit substantial improvement in left ventricular function and recover almost completely as compared with acute myocarditis (AM) once they survive the acute phase.^[6]^ However, the outcome could not be predicted with certainty in the early stage of hospitalization.^[7–9]^ Searching tools to assist diagnosis, monitor disease activity and evaluate prognosis of FM has been one of the most active areas of investigation in myocarditis.

Histological evidence of inflammatory cell infiltrates by endomyocardial biopsy (EMB) is the gold criteria to diagnose myocarditis.^[10]^ In comparison, FM is not a histological or an immunohistological diagnosis.^[11,12]^ The amount of infiltrating cells in biopsy specimen is not equivalent of FM and has no diagnostic and prognostic relevance.^[13]^ Moreover, EMB is rarely performed clinically due to the limited ability in most centres, or the procedural risk particularly in FM. Tissue characterization by cardiac magnetic resonance (CMR) is becoming a routine and mostly used non-invasive imaging tool to confirm myocarditis recently.^[14]^ The magnitude of necrosis and/or oedema by late gadolinium enhancement (LGE) and T1/T2 mapping on CMR can provide incremental prognostic information in AM.^[15–17]^ However, CMR is limited during the early stage of FM, particularly when circulatory supports are used. Thus, it is not a good candidate to detect inflammatory myocardial injuries in the early stage of FM and monitor their activity during the course of FM.

Echocardiography is one of the routine examinations and can be performed on bedside as needed during the course of FM. Conventional echocadiography provides limited information other than left ventricular (LV) function. 2D speckle tracking echocardiography (2D STE) is an evolving method to determine the LV global and regional function quantitatively and is more sensitive than conventional echocardiographic parameters. Recent studies showed that strain imaging by 2D-STE is correlated well with cell infiltration in tissue specimen obtained by EMB and oedema detected by CMR in AM.^[18–20]^ Therefore, we propose that strain by 2D-STE may be used as an additional tool to assist diagnosis and provide prognostic information in FM.

This study included 18 survived patients with clinical diagnosis of FM by typical clinical characteristics, elevated necrosis biomarkers and positive “Lake-Louise” CMR criteria. Sequential echocardiographic images were performed and strains by 2D STE were analysed at admission and before discharge. CMR imaging was performed before discharge. The correlations of strains during the course with tissue characteristics by CMR were assessed. We aimed to analyse whether strain imaging by 2D STE can monitor myocardial injuries during the course of FM and evaluate its usefulness in FM.

## 2 Materials and methods

### 2.1 Study population and design

**Figure 1:**
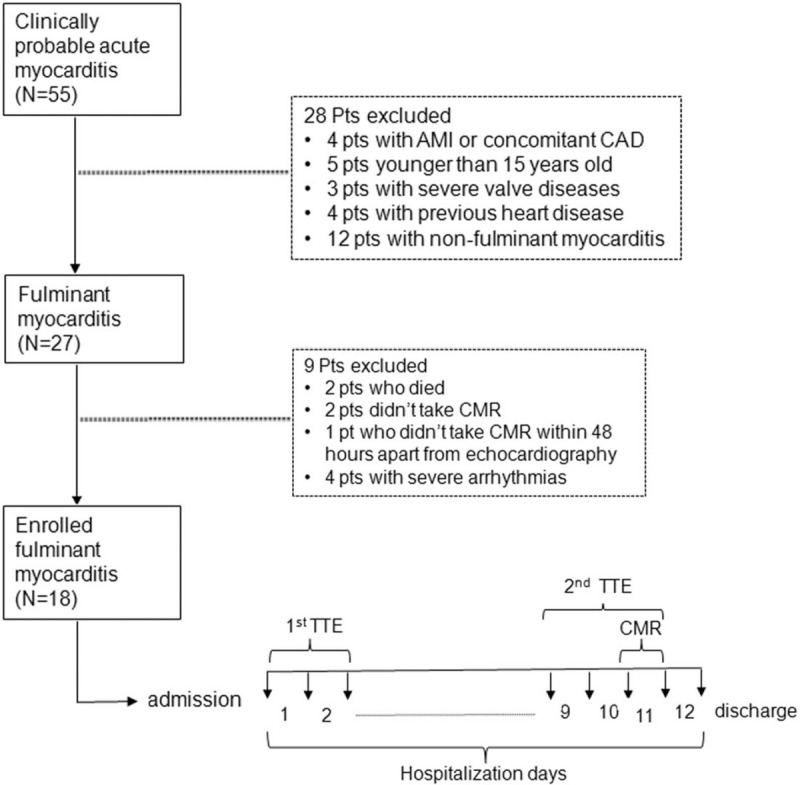
Flow chart of enrolling patients and protocol of performing TTE and CMR. 55 patients with probable myocarditis were screened initially and the final study population consisted of 18 patients. The mean hospitalization period was 12 ± 4 days. The CMR was performed before hospital discharge and at mean 11 ± 4 days after admission. The first TTE was performed at day 1 or 2 of admission. The second TTE was performed before discharge as well at no more than 48 hours apart from CMR. TTE = transthoracic echocardiography.

Tongji hospital is one of the most important AM and FM centre in China and most FM patients in the region around were admitted in our hospital. Fifty-five patients from June 30, 2017 to Apr 30, 2019 with probable diagnosis of acute myocarditis as suggested by S.Sagar^[21]^ were prospectively screened. Coronary angiography was performed in patients older than 25 years to rule out acute myocardial infarction and concomitant coronary artery disease. Other exclusion criteria were: severe valve diseases, subjects < 15 years of age, previous heart disease history (Fig. 1). Of patients with myocarditis, 27 patients were diagnosed as FM by meeting the following criteria^[22,23]^:

(1) having rapid onset of symptoms of acute heart failure within less than two days and a flu-like prodrome;
(2) having severe hemodynamic compromise that needed high doses of vasopressors (≥5 μg/kg/min of dopamine or dobutamine);
(3) mechanical circulatory supports, such as intra-aortic balloon pumps and/ or venoarterial extracorporeal membrane oxygenation, were used in the early phase.

Twelve patients who didn’t met the criteria were considered as non-fulminant myocarditis and were excluded from the study.

We subsequently excluded the following patients:

(1) 2 patients who died;
(2) 2 patients who didn’t take CMRs before discharge or didn’t meet positive “Lake-Louise” criteria of myocarditis^[24]^;
(3) 1 patient who underwent CMRs in more than 48 hours apart from the second echocardiography scans;
(4) 4 patients with poor echocardiographic images or the presence of arrhythmias that interfered with strain analysis.

Thus, the final study population was consisted of 18 patients. Figure 1 showed the flow chart for enrolling patients and performing examinations of transthoracic echocardiography (TTE) and CMR.

All 18 patients received treatment which is referred to as “Life-Support-Based Comprehensive Treatment Regimen (LSBCTR)”^[25]^ that includes

(i) mechanical life support (positive pressure respiration, IABP with or without ECMO),
(ii) immunomodulation therapy using sufficient doses of glucocorticoids and immunoglobulins, and
(iii) application of neuraminidase inhibitors.

All procedures were in accordance with the ethical standards of the responsible committee on human experimentation (institutional and national) and with the Helsinki Declaration of 1975, as revised in 2000. Informed consent was obtained from all patients included in the study. And this study was approved by the Research Ethics Committee of Tongji Hospital.

### 2.2 Echocardiographic scans and data analysis

A Vivid E9 ultrasound scanner (GE Vingmed; Horten, Norway) was used for TTE scans. Sequential TTEs were taken at a frequency of 1 scan per 1 to 2 days from the admission till the day when the LV ejection fraction (EF) recovered to >50% before discharge. Two scans were used for the data analysis, one at admission when the lowest LV function was recorded (day 1 or 2 of admission) and the second one before discharge when the patients’ hemodynamic statuses were stable and LV function almost was recovered (LVEF > 50%). Figure 1 showed the time when the two TTE scans were performed.

LV and left atrial dimensions and LV wall thickness were measured using standard methods. LV EF was measured by the modified biplane Simpson's method. Global longitudinal strains (GLS) were obtained by 2D STE. 2D greyscale images of standard four-chamber, two-chamber and three chamber views with optimized focus on the LV were used for myocardial strain analysis as described previously.^[26]^ Briefly, three cardiac cycles of each apical view were recorded at frame rate ≥60 frames/s. Duration of systole was defined in the five-chamber apical view by marking the aortic valve opening and closure from the continuous wave Doppler curve. An experienced investigator who was unaware of the patients’ clinical information and CMR data conducted the strain analysis off-line by using EchoPAC software (version:113, 2017; GE Vingmed; Horten, Norway). The LV borderline was manually traced in each apical plane, and motion tracking was performed automatically by the software. Segmental peak systolic longitudinal strain values were averaged to achieve global GLS according to AHA 17-segment LV model.

### 2.3 CMR acquisition and analysis

CMR was performed before discharge, same as the second TTE. The time interval between CMR and the second TTE scan was within 48 hours (Fig. 1).

All patients were scanned on a 3-T Magnetic Resonance Imaging (MRI) scanner (MAGNETOM Skyra, Siemens Healthcare, Erlangen, Germany) with a 18-channel body matrix coil. The diagnosis of myocarditis was made by “Lake Louise criteria”.^[24]^ Cine images were obtained using steady state free precession sequence during breath hold in short axis and three long axis planes. The parameters were as follows: FOV, 360 × 360 mm^2^; TR/TE/flip angle, 37.7 ms/1.4 ms/55°; slice thickness, 8 mm; voxel size, 1.9 × 1.9 × 8.0 mm; bandwidth, 965 Hz/pixel. Black blood T2-weighted short tau inversion recovery MRI was performed in short axis plane covering the whole left ventricle (LV) using TR of 2 RR intervals; TE: 41ms; slice thickness, 8mm; FOV: 360 × 360 mm^2^. Native and post-contrast T1 mapping were performed using a MOLLI sequence with 5(3)3 and 4(1)3(1)2 acquisition scheme, respectively. The acquisition parameters were TR/TE/flip angle, 3.8 ms/1.2ms/35°; voxel size, 1.4 × 1.4 × 5.0 mm. T2 mapping was performed before contrast injection using a multi-echo fast spin-echo sequence with the following parameters: acquisition matrix = 206 × 256; TE = 0, 24, 55 ms; TR = 208 ms; FA = 12°; pixel size = 1.9 × 1.9 × 5 mm^2^; and slice thickness = 5 mm. Both T1 and T2 mapping were acquired in basal, mid-ventricular, and apical short-axis slices. Finally, LGE images were obtained by a 2D phase-sensitive inversion-recovery (PSIR) gradient-echo pulse sequence (TR/TE/flip angle, 5.2 ms/1.2 ms/55°; voxel size, 1.5 × 1.5 × 8.0 mm).

All CMR images were assessed offline by 2 independent readers, experienced in cardiovascular MRI (with 2–4 years of experience) and blinded to clinical and echocardiographic information, using a dedicated CMR software cvi42 (version 5.3, Circle Cardiovascular Imaging, Calgary, Canada). The hematocrit-corrected extracellular volume fraction (ECV) and global mean values were measured on pre- and post-contrast T1 mapping images. Hematocrit samples were taken on the same day of cardiac MRI scans. Global LV native T1, T2, and ECV values were used for further analysis. Myocardial edema and non-ischemic LGE lesions were qualitatively assessed by consensus from the 2 observers. For each patient, the endo- and epicardial contours of all short axis LGE images were manually delineated, then LGE mass was obtained by using a signal intensity threshold of ≥5SD above the mean signal of the remote reference myocardium.

### 2.4 Statistical analysis

Data are expressed as mean ± SD for continuous variables or numbers (percentages) for categorical variables. Continuous variables between groups were analysed by Student *t* test or Mann-Whitney *U* test according to its distribution. For paired comparisons, paired *t* test or paired sample Wilcoxon rank test were performed for continuous variables, depending on the normality of the variables. Univariate linear regression was used to determine the effect of continuous strain variables on fibrosis and oedema detected by LGE and T1/T2 mapping by CMR before discharge. Covariate was selected by clinical perspective and applied in the multivariate linear regression model. All analysis was performed using SPSS version 19.0 software (SPSS, Inc., Chicago, IL). Statistical tests were two-tailed and a *P*-value of less than 0.05 was considered statistically significant.

### 2.5 Reproducibility

To investigate inter- and intra-personal measurement reproducibility, measurements were performed off-line in all subjects by 2 independent investigators. The intra-personal agreement was measured 7 days later by the same investigator. The interclass correlation coefficients were calculated; the point estimates and the 95% confidence intervals were reported.

## 3 Results

### 3.1 Clinical characteristics

Patients clinical characteristics were presented in Table 1. The mean age was 34 ± 18 years with 8 males and 10 females. The average hospitalization time for the 18 patients was 12 ± 4 days. Both levels at admission and before discharge were described for parameters as blood pressure, heart rate and some laboratory findings. Systolic blood pressure was increased from 95 ± 12 mmHg at admission to 116 ± 8 mm Hg before discharge and heart rate was decreased from 105 ± 19 bpm to 74 ± 6 bpm, showing that the patients’ hemodynamic statuses were stabilized before discharge. Peak troponin-T value was 33345.6 ± 17000.4 pg/mL and NT-proBNP value was 13539.8 ± 12694.6 pg/ml at admission. They both significantly declined before discharge but remained elevated at values of 591.4 ± 1091.9pg/mL and 959.5 ± 779.6 pg/ml, respectively. IABP was applied in 17 patients in the first or second day of admission and ECMO was performed in 4 patients.

**Table 1:** Clinical characteristics of patients with fulminant myocarditis at admission and before discharge.

	Admission (n = 18)	Discharge (n = 18)	*P* value
Age, yr	34 ± 17	N/A	N/A
Gender, F/M	10/8	N/A	N/A
Height, cm	164 ± 6	N/A	N/A
Weight, kg	63.6 ± 11	N/A	N/A
Systolic blood pressure (mm Hg)	95 ± 12	116 ± 8	*P* < .001
Diastolic blood pressure (mm Hg)	61 ± 9	68 ± 8	.023
Heart rate (bpm)	105 ± 19	74 ± 6	*P* < .001
Temperature at admission, °C	36.6 ± 0.5	36.6 ± 0.3	.786
Laboratory findings			
C-reactive protein, mg/L	38.8 ± 44.7	12.1 ± 12	.021
Peak troponin-T, pg/mL	33345.6 ± 17000.4	591.4 ± 1091.9	*P* < .001
NT-proBNP, pg/mL	13539.8 ± 12694.6	959.5 ± 779.6	*P* < .001
ALT, U/L	330.2 ± 715.1	69.6 ± 44.5	.132
AST, U/L	471.3 ± 668.4	97.3 ± 78.1	.024
Creatinine (μmol/l)	85.2 ± 51.2	64.3 ± 12.9	.027
Lactic acid(μmol/l)	2.6 ± 1.2	N/A	N/A
Treatments in-hospital			
Methylprednisolone, n(%)	18 (100)	N/A	N/A
Gamma globulin, n(%)	18 (100)	N/A	N/A
CRRT, n(%)	15 (83)	N/A	N/A
IABP, n(%)	17 (94)	N/A	N/A
v-a ECMO, n(%)	4 (25)	N/A	N/A

### 3.2 Echocardiographic features at admission and before discharge

**Figure 2:**
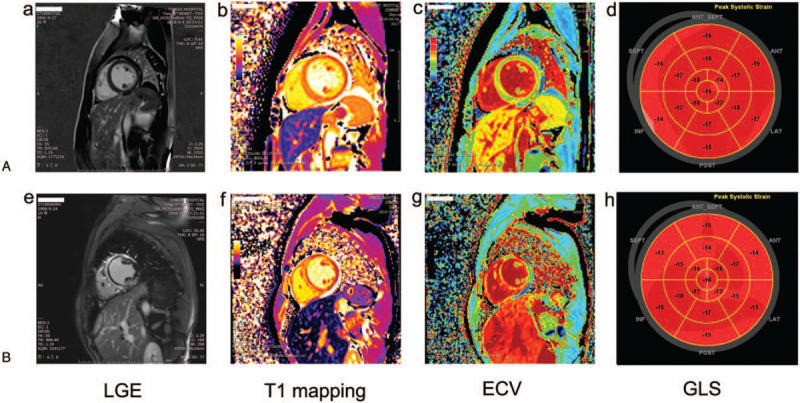
Representative CMR images and the longitudinal strains by 2D-STE presented as ‘bull's eye’ displays. The figure shows LGE images (A, E), global native T1 mapping (B, F), ECV measurements (C, G) and the corresponding strain images (D, H). (A) is a patient with least amount of LGE but greater oedema (higher T1 mapping and ECV), and the GLS value is 16.4%; (B) is another patient with diffuse and a large amount of LGE (arrow heads) but lessor oedema (lower T1 mapping and ECV), and the GLS value is 15.7%.

Echocardiographic parameters at admission and before discharge were shown in Table 2. Briefly, LV chamber sizes in the end of diastole (LV end-diastolic dimensions and Left ventricular end-diastolic volume) were in normal range at admission and no significant changes were seen before hospital discharge. The wall thickness of interventricular septum thickness in diastole at admission was slightly increased than normal, and the thickened walls had no significant regression on discharge. All patients had severely impaired LVEF at admission and great improvement before hospital discharge (EF: 32 ± 14% vs 55 ± 7%, fractional shortening: 15 ± 7% vs 30 ± 6%, *P* < .001). Parallel to the recovery of LVEF, GLS values were increased significantly from 9.6 ± 4.7% at admission to 17.2 ± 3.1% before discharge (*P* < .001). The representative images of the longitudinal strains before discharge presented as ‘bull's eye’ in two patients were shown in Figure 2.These findings indicate that severely damaged LV function of the FM patients in the study can improve significantly on hospital discharge.

**Table 2:** Echocardiographic parameters at admission and before discharge.

Parameters	Admission (n = 18)	Discharge (n = 18)	*P* value
IVS diastolic (cm)	1.07 ± 0.21	1.05 ± 0.19	.683
IVS systolic (cm)	1.31 ± 0.19	1.34 ± 0.14	.617
LVEDD(cm)	4.42 ± 0.48	4.4 ± 0.60	.975
LVESD(cm)	3.58 ± 0.55	3.23 ± 0.60	.081
LVEDV, mL	105.11 ± 33.66	108.76 ± 32.47	.746
LVESV, mL	70.67 ± 26.29	48.94 ± 19.67	.009
LA diameter, cm	2.98 ± 0.42	2.86 ± 0.42	.389
EF, %	32 ± 14	55 ± 7	<.001
FS, %	15 ± 7	30 ± 6	<.001
Global longitudinal strain, %	- 9.6 ± 4.7	-17.2 ± 3.1	<.001

### 3.3 CMR characteristics before discharge

CMRs were performed before discharge, at day 11 ± 4 of admission. Table 3 summarized the tissue characteristics detected by CMR, including LGE, global native T1 and T2 relaxation time, and ECV. LV EF had greatly improved to 53 ± 8% before discharge. All 18 (100%) patients had evidence of non-ischemic LGE that is subepicardial or midwall LGE and LGE accounted for 31.7 ± 22.8% of the LV mass. Global native T1 and T2 relaxation time and ECV measurement were 1408.3 ± 88.3ms, 46.56 ± 5.23ms and 0.35 ± 0.09, respectively. Figure 2 was representative CMR images in 2 patients showing the presence of LGE in a non-ischemic pattern and measurements of global native T1 mapping and ECV. Global native T1 and T2 measurements as well as ECV values in normal subjects at 3T CMR by our group were 1213 ± 36ms, 37 ± 1ms and 0.22 ± 0.03, respectively (unpublished data in our lab). All these three measurements were greatly elevated in comparison with normal subjects. In addition, patients with FM had presence of both fibrosis and oedema together, but the extent of fibrosis and oedema varied greatly. Figure 2A was a patient with least amount of LGE but greater oedema (higher T1 mapping and ECV). In comparison, Figure 2B showed a patient with a large amount of LGE but lessor oedema (lower T1 mapping and ECV).

**Table 3:** CMR parameters before discharge.

Parameters	Discharge (n = 18)
CMR performed-no. (%)	18 (100)
Time to CMR from admission-days	11 ± 4
Interventricular septal thickness (cm)	1.03 ± 0.17
LV EF, %	53 ± 8
Diffuse LGE-no. (%)	18 (100)
LGE mass (g)	22.6 ± 20.6
LGE mass %	31.7 ± 22.8
T1 mapping (ms)	1408.3 ± 88.3
ECV	0.35 ± 0.09
T2 mapping (ms)	46.56 ± 5.23

### 3.4 Correlation of GLS with tissue characteristics by CMR

**Figure 3:**
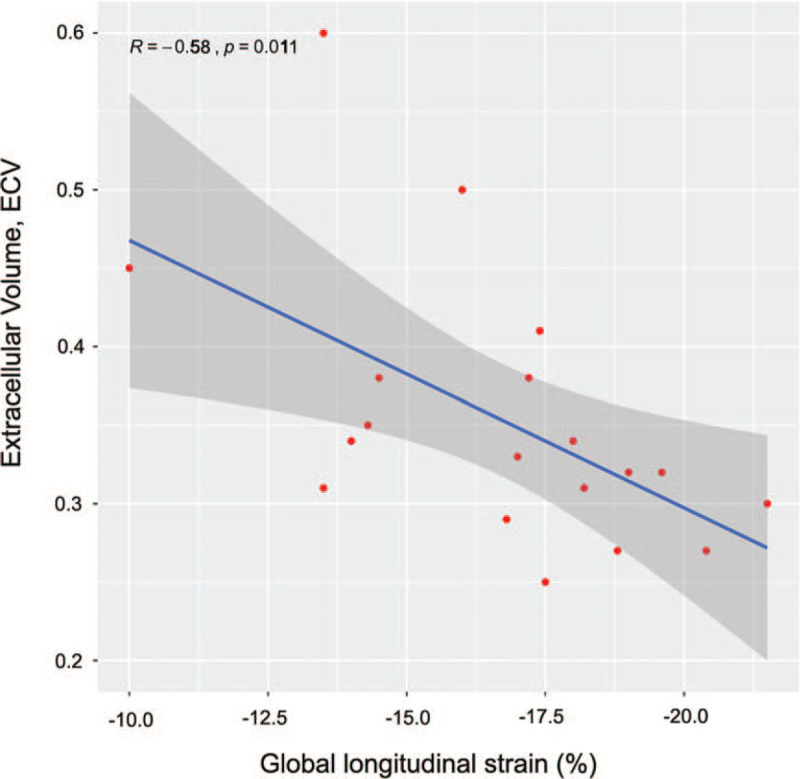
Correlation between GLS before discharge and ECV by CMR. CMR: cardiac magnetic resonance.

We first performed univariate analysis between CMR parameters with GLS derived from second TTE scan before discharge. CMR and the second TTE scan were both performed before discharge which was in the same recovery stage during the course of FM. We observed that GLS before discharge was correlated significantly with ECV (***r*** = -0.58, *P* = .011) (Table 4 and Fig. 3) and with global native T1 relaxation time *(**r*** = -0.574, *P* = .013) (Table 4). However, it wasn’t correlated with global native T2 relaxation time (***r*** = -0.31, *P* = .211) and not with LGE mass (***r*** = 0.084, *P* = .743) as well (Table 4). After correcting for systolic blood pressure, diastolic blood pressure, NT-proBNP, left ventricular EF, and troponin-T in the multivariate analysis, we found a significant correlation between GLS before discharge and ECV by CMR (Table 5). As shown in figure 2, the GLS value in patient B who had relatively diffuse LGE was not greater than patient A who had least LGE. Therefore, GLS may reflect the total contribution of both fibrosis and oedema on cardiac function in FM.

**Table 4:** The liner associations between values taken by CMR and GLS.

	GLS before discharge		GLS at admission		GLS changes	
	*r*	*P*-value	*r*	*P*-value	*r*	*P*-value
LGE mass	0.084	.743	0.292	.239	−0.196	.437
LGE mass %	0.052	.838	0.28	.261	−0.205	.414
T1 mapping	−0.574	.013	−0.437	.07	0.149	.556
ECV	−0.582	.011	−0.043	.865	−0.247	.323
T2 mapping	−0.31	.211	−0.421	.082	0.214	.393
EF at admission	0.369	.132	0.816	<.001	−0.587	.01
EF before discharge	0.613	.007	0.164	.516	0.158	.532

**Table 5:** Univariate and multivariate analysis for the predictor of ECV oedema amount by CMR.

	Univariate analysis *P*-value	Multivariate analysis^a^*P*-value
Age, years	0.178	–
Systolic blood pressure, mmHg	0.204	–
Diastolic blood pressure, mmHg	0.054	–
Heart rate, bpm	0.515	–
NT-proBNP, ng/L	0.019	0.031
Left ventricular EF, %	0.119	–
Peak troponin-T, mg/L	0.085	–
GLS before discharge,%	0.011	0.005

We next compared CMR parameters with baseline GLS values. We found that there was no correlation between GLS at admission and CMR parameters before discharge, including global native T1 and T2 mapping, ECV as well as LGE (Table 4). GLS at admission also wasn’t correlated with LV EF before discharge (Table 4). Finally, delta GLS, defined as GLS changes between admission and discharge, wasn’t correlated with all CMR parameters (Table 4). In terms of serum markers, NT-proBNP level at admission was correlated with ECV (***r*** = 0.546, *P* = .019) and T1 (***r*** = 0.625, *P* = .006) (Tables 5 and 6). In contrast, peak values of cardiac tropnin-T level at admission was correlated with LGE mass (***r*** = 0.515, *P* = .029) but not T1/T2 mapping and ECV.

**Table 6:** Univariate analysis for the clinical predictor of T 1 mapping by CMR.

	T 1 mapping	
	*r*	*P*-value
Systolic blood pressure, mmHg	−0.475	0.051
Diastolic blood pressure, mmHg	−0.188	0.455
Heart rate, bpm	0.345	0.16
NT-proBNP, ng/L	0.625	0.006
Left ventricular EF, %	−0.5	0.034
Peak troponin-T, mg/L	0.425	0.078

### 3.5 Reproducibility

There were good intra-observer and inter-observer correlation for strain measurement. Interclass correlation coefficients was 0.90 (95% CI 0.85–0.93) for inter-observer agreement and 0.94 (95% CI 0.90–0.97) for intra-observer agreement.

## 4 Discussion

The present study represents an assessment of myocardial deformation changes by speckle tracking echocardiography and an evaluation of myocardial oedema and/or fibrosis by CMR in survived FM patients. Particularly, the myocardial strains during acute and early recovery stages of FM were compared with tissue characteristics by CMR. The major findings of the study were that

(1) FM patients who survived the acute phase had recovery of cardiac function in a short term,
(2) FM patients in the early recovery stage had different tissue patterns with varied amount of oedema and fibrosis,
(3) GLS can be a great indicator to monitor the course of myocardial inflammation in FM, and
(4) the initial lower GLS does not reflect poor in-hospital recovery of cardiac function in FM.

The in-hospital outcome of FM is poor with mostly reported survival rates of between 46% to 75.5%.^[1–4]^ Recently, a group from Japan reported much higher survival rate of 83.3% was with mechanical circulatory supports.^[27]^ A multi-centre study led by our group reported even lower in-hospital mortality of 3.7% with a LSBCTR.^[28]^ From the point of LV function, a study showed that the patients with FM had more severely impaired LVEF at admission and had steep improvement during hospitalization compared with non-fulminant myocarditis.^[1]^ Our study consisted of 18 survived patients with FM who all used LSBCTR and had clinical recovery in average 12 hospitalization days. They all had very severe LV dysfunction at admission with dramatically lower levels of EF and GLS but presented with rapid improvement before discharge. This finding provides new evidences that FM patients can have short term recovery of cardiac function once they survive the acute phase after appropriate treatments. These patients are being followed up in our centre to evaluate their long-term outcome.

The Consensus Criteria for CMR in Myocardial Inflammation (“Lake Louise Criteria”) published in 2009 proposed 3 diagnostic targets-edema, hyperemia, and necrosis, which derived respectively from signal intensity assessment in T2-weighted, early gadolinium enhancement (EGE) and LGE CMR images.^[16,29,30]^ While the criteria provides good overall diagnostic performance, novel CMR mapping techniques as T1 and T2 mapping, and ECV were reported to have additional values in patients with suspected myocarditis. LGE is known to reflect focal necrosis/fibrosis, usually indicating irreversible or persistent myocardial injury.^[24]^ Elevated T1 mapping and ECV represent both acute inflammation/oedema and irreversible myocardial fibrosis, with T1 more sensitive to acute inflammation.^[16,31,32]^ T2 mapping has the best value to differentiate acute from chronic myocarditis, representing mainly acute inflammation or reversible oedema.^[6,16,30]^ CMR in this study was performed before discharge and therefore reflected tissue characteristics in the early recovery stage of FM. The results showed that all patients had focal LGE in a non-ischemic pattern and had elevated T1/T2 mapping and ECV as well. It indicates that myocardial inflammation may be still ongoing other than chronic fibrosis in this stage. However, the extent of fibrosis and oedema varied greatly in individual patients. Some patients had mainly fibrosis and others had predominantly oedema. Follow-up study is needed to investigate the effects of these different tissue patterns on the long-term outcome of FM.

Compared with CMR, the portability of TTE makes it a great imaging tool in the management of FM. However, traditional echocardiography provides least information other than LV function in myocarditis. In contrast, strain analysis by 2D STE is more sensitive in evaluating LV function in AM.^[19]^ Studies in AM had demonstrated that there was significant correlation between strains by 2D-STE and oedema by CMR.^[19,20]^ The other study demonstrated a positive correlation between amplitude of strain and delayed enhancement on CMR in patients with AM.^[33]^ To investigate the utility of myocardial strain in FM, we did the correlation analysis between CMR parameters and GLS from the same recovery stage of FM first. Our study showed that GLS was correlated significantly with CMR ECV but not with LGE and T2, which was different from the previous report in AM.^[33]^ The reason why the finding from FM is different from that of AM may because of the proportion of fibrosis and oedema. Strain is actually a measure of regional myocardial deformation and either fibrosis/necrosis or oedema will affect strain measurement. In the AM, oedema was the mainly changes of the myocardium. In early recovery stage of FM, fibrosis/necrosis and oedema were both pronounced as shown in our study. They two together contribute to the reduction of myocardial deformation or strain, but not fibrosis/necrosis or oedema alone. In addition, the different degree of inflammatory myocardial injuries may also cause the difference correlation of GLS and CMR in AM and FM. CMR parameters in the early phase of FM were not obtained in the study, and the correlation of GLS with CMR in the acute phase was not known. In summary, the good correlation of GLS with fibrosis and oedema detected by CMR and the portability of TTE make 2D STE a useful tool to monitor myocardial inflammatory injuries in the clinical management of FM.

The present study also tried to evaluate whether strains by 2D STE during the early phase can predict in-hospital outcome or LV function recovery in FM. We thus correlated GLS in the early phase to CMR in the recovery stage of FM. The results found that the initial GLS did not correlate with the extent of LGE or oedema by T1/T2 mapping by CMR. In other words, the initial lower GLS did not predict more severe necrosis and/or oedema and thus poor cardiac function in the early recovery stage of FM by our study. This finding is different from the previous reports in AM. A retrospective study in paediatric myocarditis found that the presence of LVEF < 30% on admission was the major predictor for poor outcomes.^[7]^ Another report also found that the initial LV EF was the only independent predictor of outcome in AM.^[34]^ Our study proved that the baseline GLS is not a predictor of the early recovery in FM.

## 5 Limitations

The present study has several limitations. First, the study was conducted in a single centre and the patients enrolled were in relatively small numbers. In addition, patients with high-degree heart block or irregular rhythm who are not rare in the population of FM and patients who died were excluded from the study, which can cause selective bias. Second, invasive EMB that is considered as the gold standard of myocarditis was not performed in the study. Third, the CMRs were performed only in the recovery stage of FM. The tissue characteristics of CMR in the early phase of FM and its correlation with GLS were not assessed in the present study. However, CMR is hardly performed in the acute phase of FM when patients’ statuses are not stable. Finally, fibrosis and oedema have different pathological and prognostic significance. Follow-up studies are needed to evaluate the long-term outcomes of FM patients, including death, heart transplantation, LV EF reduction and LV dilatation. We are aimed to define the significance of different CMR characteristics and/or myocardial strains for predicting the long-term outcomes of FM.

## 6 Conclusions

Myocardial deformation or strain analysis by 2D STE appears to be very informative in FM and may emerge as a new tool to monitor inflammatory myocardial injuries during the course of FM. FM in the early recovery stage has the presence of both chronic fibrosis and acute oedema as detected by CMR, which have good correlation with GLS by 2D-STE at the same stage. Moreover, GLS at admission shows no correlation with the extent of fibrosis and oedema at discharge by CMR and can’t predict the early recovery of myocardial inflammation and cardiac function in FM.

## Author contributions

**Conceptualization:** Houjuan Zuo, Hong Wang, Dao Wen Wang.

**Data curation:** Houjuan Zuo, Haojie Li, Rui Li, Jiangang Jiang, Hong Wang.

**Formal analysis:** Houjuan Zuo, Haojie Li, Rui Li.

**Funding acquisition:** Houjuan Zuo, Hong Wang.

**Investigation:** Houjuan Zuo, Haojie Li, Jiangang Jiang, Chenze Li.

**Methodology:** Fei Ma, Jiangang Jiang.

**Project administration:** Liming Xia, Dao Wen Wang.

**Resources:** Hong Wang.

**Software:** Houjuan Zuo, Rui Li, Fei Ma, Chenze Li, Liming Xia.

**Supervision:** Jiangang Jiang, Chenze Li, Liming Xia, Hong Wang, Dao Wen Wang.

**Validation:** Chenze Li, Hong Wang, Dao Wen Wang.

**Visualization:** Fei Ma.

**Writing – original draft:** Houjuan Zuo, Hong Wang.

**Writing – review & editing:** Hong Wang, Dao Wen Wang.

## References

[1] Ammirati E Cipriani M Lilliu M. Survival and left ventricular function changes in fulminant versus nonfulminant acute myocarditis. Circulation 2017;136:529–45. 28576783 10.1161/CIRCULATIONAHA.117.026386

[2] Chen YS Yu HY Huang SC. Extracorporeal membrane oxygenation support can extend the duration of cardiopulmonary resuscitation. Crit Care Med 2008;36:2529–35. 18679121 10.1097/CCM.0b013e318183f491

[3] Chung SY Sheu JJ Lin YJ. Outcome of patients with profound cardiogenic shock after cardiopulmonary resuscitation and prompt extracorporeal membrane oxygenation support. A single-center observational study. Circ J 2012;76:1385–92. 22447007 10.1253/circj.cj-11-1015

[4] Matsuura H Ichida F Saji T. Clinical features of acute and fulminant myocarditis in children- 2nd nationwide survey by japanese society of pediatric cardiology and cardiac surgery. Circ J 2016;80:2362–8. 27725476 10.1253/circj.CJ-16-0234

[5] Ammirati E Veronese G Brambatti M. Fulminant versus acute nonfulminant myocarditis in patients with left ventricular systolic dysfunction. J Am Coll Cardiol 2019;74:299–311. 31319912 10.1016/j.jacc.2019.04.063

[6] Felker GM Boehmer JP Hruban RH. Echocardiographic findings in fulminant and acute myocarditis. J Am Coll Cardiol 2000;36:227–32. 10898439 10.1016/s0735-1097(00)00690-2

[7] Rodriguez-Gonzalez M Sanchez-Codez MI Lubian-Gutierrez M. Clinical presentation and early predictors for poor outcomes in pediatric myocarditis: a retrospective study. World J Clin Cases 2019;7:548–61. 30863755 10.12998/wjcc.v7.i5.548 PMC6406197

[8] Sawamura A Okumura T Hirakawa A. Early prediction model for successful bridge to recovery in patients with fulminant myocarditis supported with percutaneous venoarterial extracorporeal membrane oxygenation- insights from the change pump study. Circ J 2018;82:699–707. 29081472 10.1253/circj.CJ-17-0549

[9] Sawamura A Okumura T Ito M. Prognostic value of electrocardiography in patients with fulminant myocarditis supported by percutaneous venoarterial extracorporeal membrane oxygenation- analysis from the change pump study. Circ J 2018;82:2089–95. 29863096 10.1253/circj.CJ-18-0136

[10] Caforio AL Pankuweit S Arbustini E. Current state of knowledge on aetiology, diagnosis, management, and therapy of myocarditis: a position statement of the european society of cardiology working group on myocardial and pericardial diseases. Eur Heart J 2013;34:2636–48. 23824828 10.1093/eurheartj/eht210

[11] Aretz HT. Myocarditis: the dallas criteria. Hum Pathol 1987;18:619–24. 3297992 10.1016/s0046-8177(87)80363-5

[12] Cooper LT Baughman KL Feldman AM. The role of endomyocardial biopsy in the management of cardiovascular disease: A scientific statement from the american heart association, the american college of cardiology, and the european society of cardiology. Circulation 2007;116:2216–33. 17959655 10.1161/CIRCULATIONAHA.107.186093

[13] Maisch B Noutsias M Ruppert V. Cardiomyopathies: classification, diagnosis, and treatment. Heart Fail Clin 2012;8:53–78. 22108727 10.1016/j.hfc.2011.08.014

[14] Biesbroek PS Hirsch A Zweerink A. Additional diagnostic value of cmr to the european society of cardiology (esc) position statement criteria in a large clinical population of patients with suspected myocarditis. Eur Heart J Cardiovasc Imaging 2018;19:1397–407. 29186442 10.1093/ehjci/jex308

[15] Ammirati E Moroni F Sormani P. Quantitative changes in late gadolinium enhancement at cardiac magnetic resonance in the early phase of acute myocarditis. Int J Cardiol 2017;231:216–21. 27913009 10.1016/j.ijcard.2016.11.282

[16] Bohnen S Radunski UK Lund GK. Tissue characterization by t1 and t2 mapping cardiovascular magnetic resonance imaging to monitor myocardial inflammation in healing myocarditis. Eur Heart J Cardiovasc Imaging 2017;18:744–51. 28329275 10.1093/ehjci/jex007

[17] Grani C Eichhorn C Biere L. Prognostic value of cardiac magnetic resonance tissue characterization in risk stratifying patients with suspected myocarditis. J Am Coll Cardiol 2017;70:1964–76. 29025553 10.1016/j.jacc.2017.08.050 PMC6506846

[18] Escher F Kasner M Kuhl U. New echocardiographic findings correlate with intramyocardial inflammation in endomyocardial biopsies of patients with acute myocarditis and inflammatory cardiomyopathy. Mediators Inflamm 2013;2013:875420. 23576857 10.1155/2013/875420 PMC3616345

[19] Logstrup BB Nielsen JM Kim WY. Myocardial oedema in acute myocarditis detected by echocardiographic 2d myocardial deformation analysis. Eur Heart J Cardiovasc Imaging 2016;17:1018–26. 26588987 10.1093/ehjci/jev302

[20] Luetkens JA Schlesinger-Irsch U Kuetting DL. Feature-tracking myocardial strain analysis in acute myocarditis: diagnostic value and association with myocardial oedema. Eur Radiol 2017;27:4661–71. 28500369 10.1007/s00330-017-4854-4

[21] Sagar S Liu PP Cooper LT Jr. Myocarditis. Lancet 2012;379:738–47. 22185868 10.1016/S0140-6736(11)60648-X PMC5814111

[22] Maisch B Ruppert V Pankuweit S. Management of fulminant myocarditis: a diagnosis in search of its etiology but with therapeutic options. Curr Heart Fail Rep 2014;11:166–77. 24723087 10.1007/s11897-014-0196-6

[23] McCarthy RE 3rd Boehmer JP Hruban RH. Long-term outcome of fulminant myocarditis as compared with acute (nonfulminant) myocarditis. N Engl J Med 2000;342:690–5. 10706898 10.1056/NEJM200003093421003

[24] Friedrich MG Sechtem U Schulz-Menger J. Cardiovascular magnetic resonance in myocarditis: a jacc white paper. J Am Coll Cardiol 2009;53:1475–87. 19389557 10.1016/j.jacc.2009.02.007 PMC2743893

[25] Wang D Li S Jiang J. Chinese society of cardiology expert consensus statement on the diagnosis and treatment of adult fulminant myocarditis. Sci China Life Sci 2019;62:187–202. 30519877 10.1007/s11427-018-9385-3 PMC7102358

[26] Delgado V Mollema SA Ypenburg C. Relation between global left ventricular longitudinal strain assessed with novel automated function imaging and biplane left ventricular ejection fraction in patients with coronary artery disease. J Am Soc Echocardiogr 2008;21:1244–50. 18992675 10.1016/j.echo.2008.08.010

[27] Saito S Toda K Miyagawa S. Diagnosis, medical treatment, and stepwise mechanical circulatory support for fulminat myocarditis. J Artif Organs 2018;21:172–9. 29236180 10.1007/s10047-017-1011-4

[28] Li S Xu S Li C. A life support-based comprehensive treatment regimen dramatically lowers the in-hospital mortality of patients with fulminant myocarditis: a multiple center study. Sci China Life Sci 2019;62:369–80. 30850929 10.1007/s11427-018-9501-9

[29] Dabir D Vollbrecht TM Luetkens JA. Multiparametric cardiovascular magnetic resonance imaging in acute myocarditis: a comparison of different measurement approaches. J Cardiovasc Magn Reson 2019;21:54. 31462282 10.1186/s12968-019-0568-x PMC6714458

[30] Von Knobelsdorff-Brenkenhoff F Schuler J Doganguzel S. Circ Cardiovasc Imaging 2017;10:e005242. 28213448 10.1161/CIRCIMAGING.116.005242

[31] Hinojar R Foote L Arroyo Ucar E. Native t1 in discrimination of acute and convalescent stages in patients with clinical diagnosis of myocarditis: a proposed diagnostic algorithm using cmr. JACC. JACC Cardiovasc Imaging 2015;8:37–46. 25499131 10.1016/j.jcmg.2014.07.016

[32] Radunski UK Lund GK Stehning C. Cmr in patients with severe myocarditis: diagnostic value of quantitative tissue markers including extracellular volume imaging. JACC. Cardiovascular imaging 2014;7:667–75. 24954462 10.1016/j.jcmg.2014.02.005

[33] Leitman M Vered Z Tyomkin V. Speckle tracking imaging in inflammatory heart diseases. Int J Cardiovasc Imaging 2018;34:787–92. 29181826 10.1007/s10554-017-1284-y

[34] Sanguineti F Garot P Mana M. Cardiovascular magnetic resonance predictors of clinical outcome in patients with suspected acute myocarditis. J Cardiovasc Magn Reson 2015;17:78. 26318624 10.1186/s12968-015-0185-2 PMC4553007

